# Supplementary figures and images for: The expression of MIR125B transcripts and bone phenotypes in Mir125b2-deficient mice
Source: PLoS One. 2024 Jul 8;19(7):e0304074. doi: 10.1371/journal.pone.0304074 (PMC11230526; doi:10.1371/journal.pone.0304074)

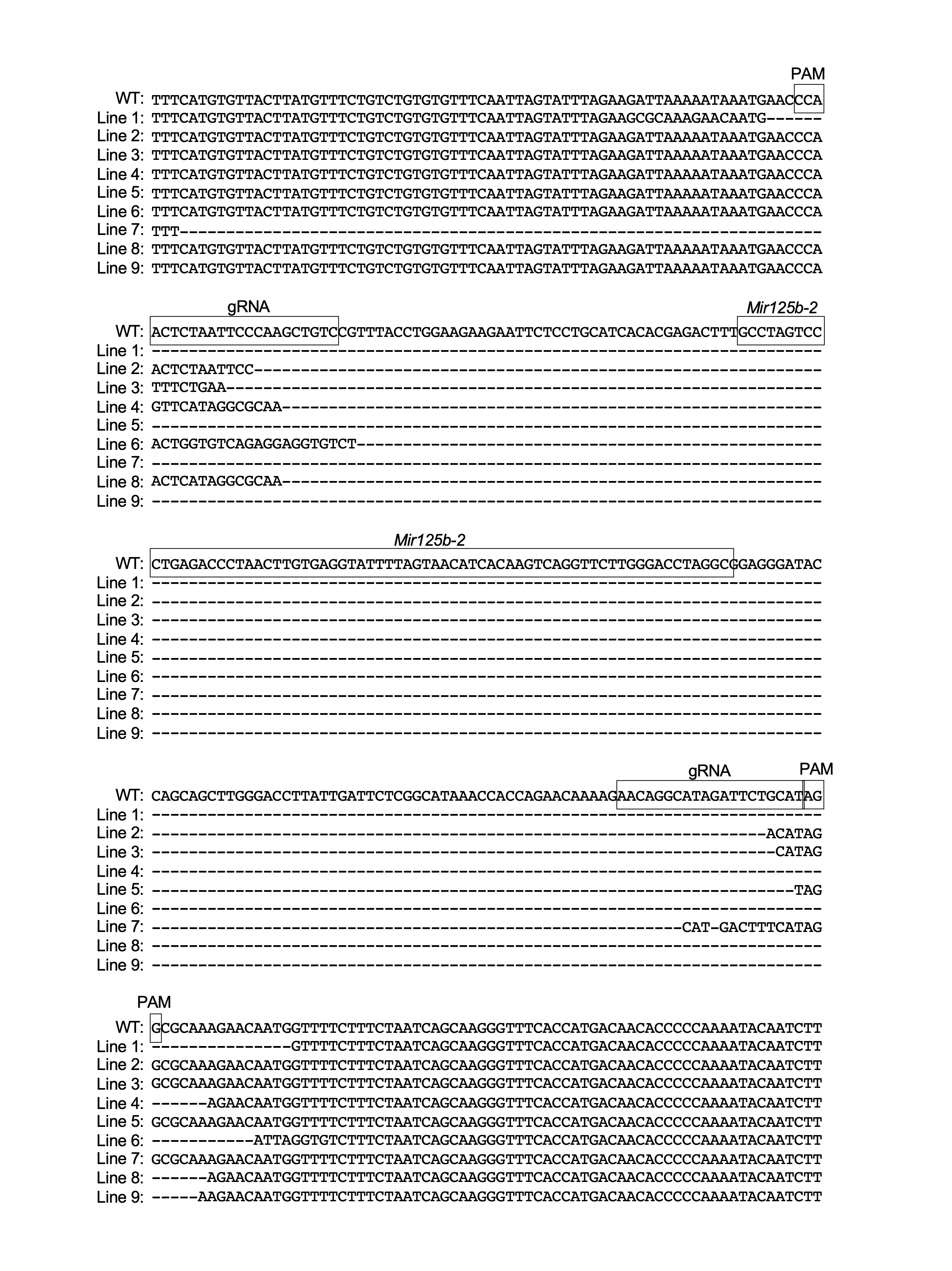

Supplement: S1 Fig — The top line indicates a wild-type mouse sequence (UCSC Genome Browser, https://genome.ucsc.edu/) as a reference, and the others are sequences from F0 mice in Line 1−Line 9. The sequences of the Mir125b2 precursor, PAM, and gRNA are enclosed within the frame. (TIFF) [file pone.0304074.s001.tiff]

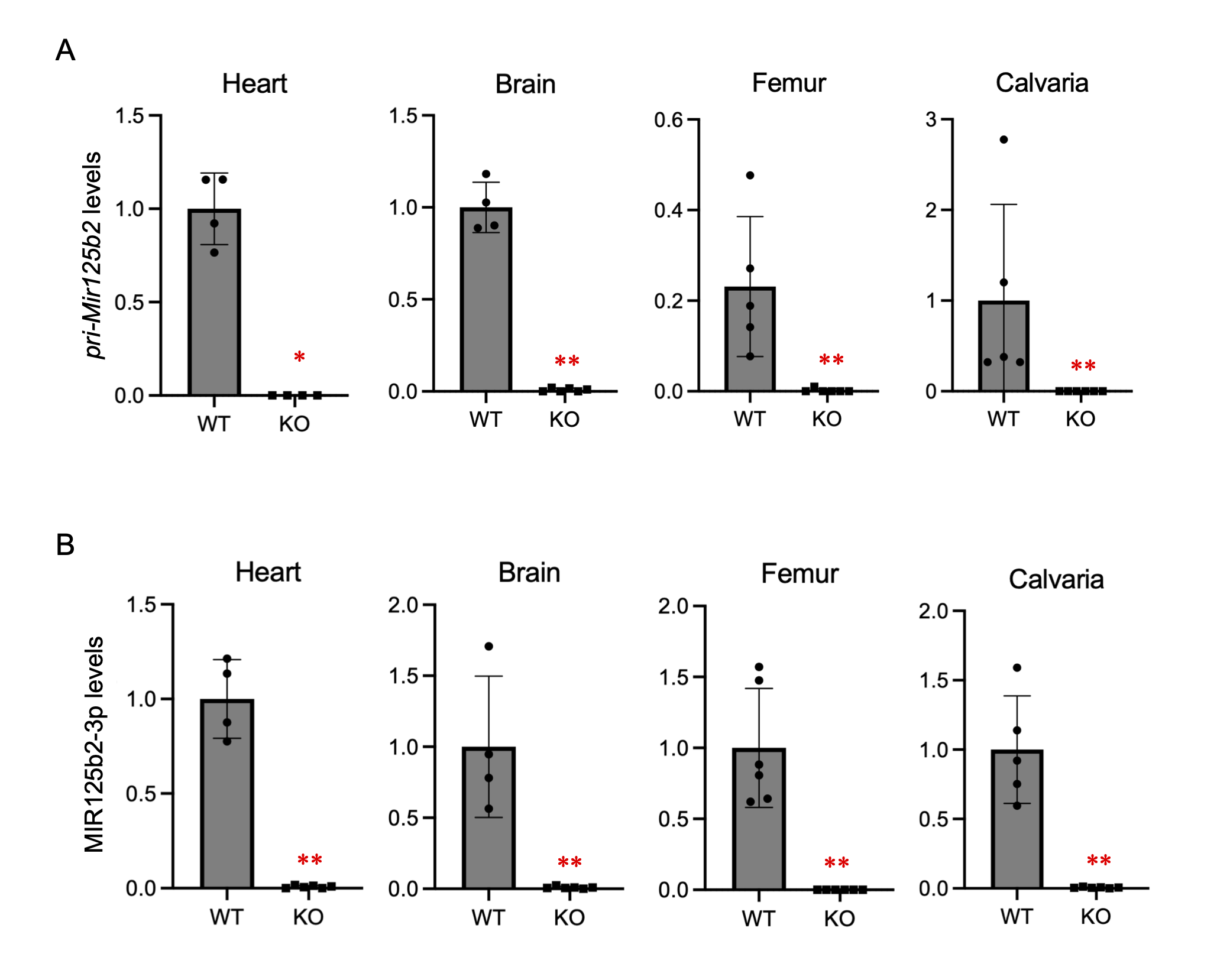

Supplement: S2 Fig — Relative levels of pri-Mir125b2 (A) and MIR125B2-3p (B) in the heart, brain, femurs, and calvaria of 12-week-old male mice. The mean values of the WT groups were set to 1.0. Actb (A) and Rnu6 (B) were used as internal controls. n = 4−6. *, p < 0.05 and **, p < 0.01 versus WT mice. (TIFF) [file pone.0304074.s002.tiff]

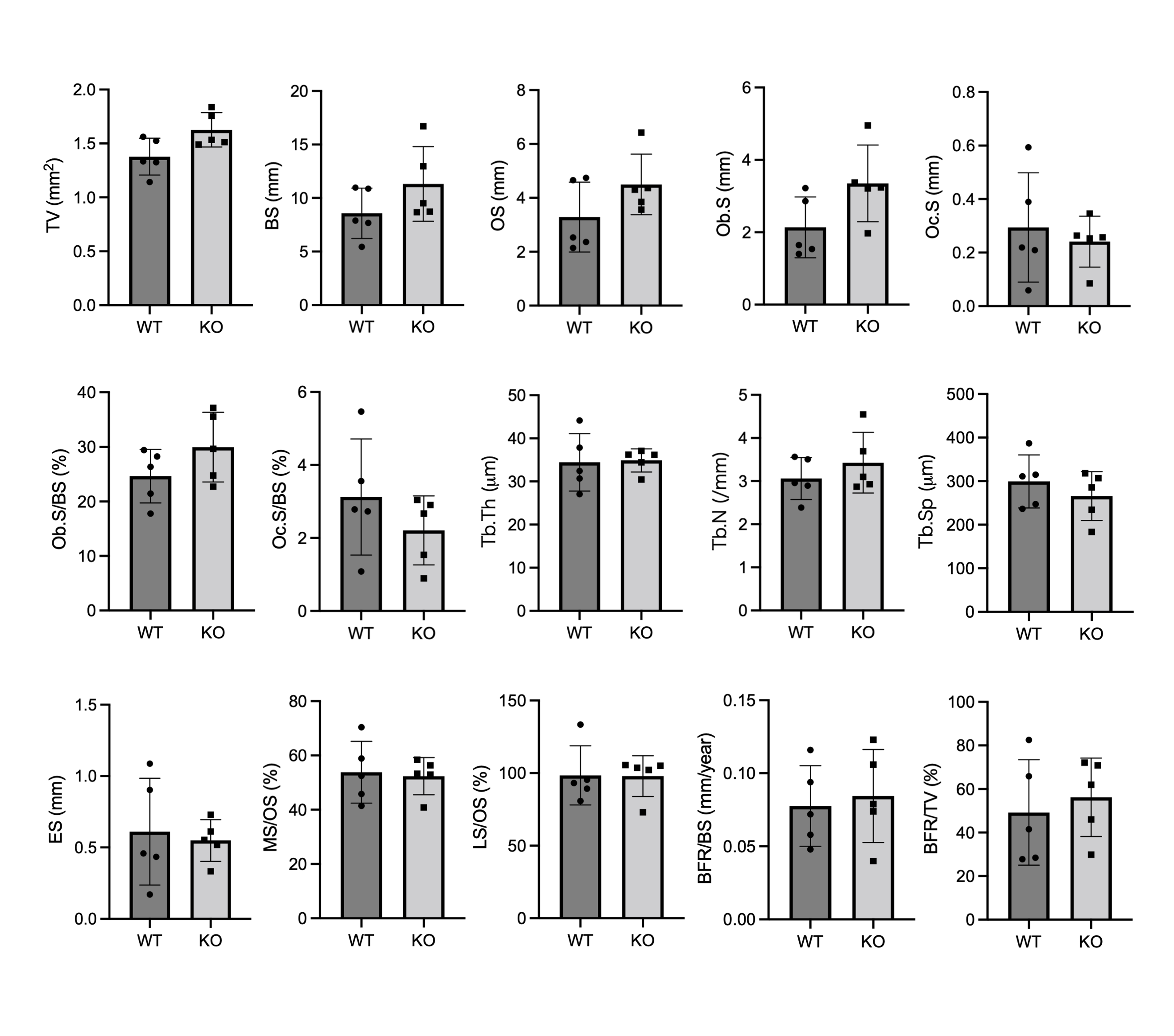

Supplement: S3 Fig — See also Fig 4(C). TV, tissue volume; BS, bone surface; OS, osteoid surface; Ob.S, osteoblast surface; Oc.S, osteoclast surface; Ob.S/BS, osteoblast surface/bone surface; Oc.S/BS, osteoclast surface/bone surface; Tb.Th, trabecular thickness; Tb.N, trabecular number; Tb.Sp, trabecular separation; ES, eroded surface; MS/OS, mineralized surface/osteoid surface; LS/OS, labeled surface/osteoid surface; BFR/BS, bone formation rate/bone surface; BFR/TV, bone formation rate/tissue volume. n = 5. (TIFF) [file pone.0304074.s003.tiff]
