# Supplementary material for: The expression of MIR125B transcripts and bone phenotypes in Mir125b2-deficient mice
Source: PLoS One. 2024 Jul 8;19(7):e0304074. doi: 10.1371/journal.pone.0304074 (PMC11230526; doi:10.1371/journal.pone.0304074)
Supplement: S1 Table — (DOCX) [file pone.0304074.s004.docx]

**S1 Table. Primer sets for PCR.**

| Gene/*pri-miRNA* | Sequence | Size (bp) |
| --- | --- | --- |
| *Mir125b2* | F: CCCATTAACTGGCATATAATCCTTT  R: TCTTCATCTTAAAACACACCCAAAG | 742 |
| *pri-Mir125b2* | F: GCTGTCCGTTTACCTGGAAGA  R: GGCAAAGTCTCGTGTGATGC | 52 |
| *Actb* | F: TTTTCCAGCCTTCCTTCTTG  R: ACGGATGTCAACGTCACACT | 89 |
