# Supplementary material for: The expression of MIR125B transcripts and bone phenotypes in Mir125b2-deficient mice
Source: PLoS One. 2024 Jul 8;19(7):e0304074. doi: 10.1371/journal.pone.0304074 (PMC11230526; doi:10.1371/journal.pone.0304074)
Supplement: S2 Table — (DOCX) [file pone.0304074.s005.docx]

**S2 Table. μCT scanning and reconstruction conditions.**

| Parameter | Value |
| --- | --- |
| Scanning  Voltage (kV)  Current (mA)  Exposure (ms)  Camera binning  Frame averaging  Filter  Pixel size (mm)  Reconstruction  Smoothing  Ring artifact correction  Beam hardening correction (%) | 50  500  900  1 x 1  OFF  Al 0.5 mm  9  1  2  15 |
