# Supplementary material for: The expression of MIR125B transcripts and bone phenotypes in Mir125b2-deficient mice
Source: PLoS One. 2024 Jul 8;19(7):e0304074. doi: 10.1371/journal.pone.0304074 (PMC11230526; doi:10.1371/journal.pone.0304074)
Supplement: Attachment — Submitted filename: Response_to_Reviewers.docx [file pone.0304074.s006.docx]

Response to Reviewers

1. Please ensure that your manuscript meets PLOS ONE's style requirements, including those for file naming.

**［Response］：** Thank you for your insightful suggestion. Accordingly, the manuscript has been rechecked and the necessary changes have been made throughout the manuscript.

2. Thank you for stating the following financial disclosure:
      "This study was supported by JSPS KAKENHI Grant Number JP22K17252. Initials of author who received it is S.I. Full name of funding is Japan Society for the Promotion of Science. 
   <https://www.jsps.go.jp>"

Please state what role the funders took in the study.

**［Response］：** Thank you for your insightful advice. The funders had no role in the design of the study, so we have stated the following in the manuscript: "The funders had no role in study design, data collection and analysis, decision to publish, or preparation of the manuscript."

3. Please review your reference list to ensure that it is complete and correct. If you have cited papers that have been retracted, please include the rationale for doing so in the manuscript text, or remove these references and replace them with relevant current references. Any changes to the reference list should be mentioned in the rebuttal letter that accompanies your revised manuscript. If you need to cite a retracted article, indicate the article’s retracted status in the References list and also include a citation and full reference for the retraction notice.

**［Response］：** Thank you for your insightful suggestion. According to your suggestion, the reference list was carefully rechecked and slight formatting corrections have been made.

4. Reviewer #1: This paper investigates the role of Mir125b2 in the bone phenotype of 3 months old mice. Using CRISPR/Ca9system the authors delete Mir125b2 globally and convincingly show the deletion with mRNA. . KO male and female mice have the same weight than respective WT control and grow normally. Deletion of Mir125b2 does not cause any bone phenotype as indicated by the analysis of the tibua by micro-CT and histomorphometry in male mice The analysis is detailed and presented appropriately.

Minor issues:

The paper would benefit the review of an English professional service.

**［Response］：** Thank you for your insightful suggestion. We have revised the manuscript. In addition, the manuscript has been proofread by a native English speaker specialized in the editing of scientific manuscripts.

The absence of the bone phenotype should be confirmed in another bone such as vertebra or femur.

**［Response］：** We are grateful for your suggestions. We collected both the femur and tibia and took μCT. The ROI had to be strictly defined so as not to miss microscopic changes, we determined by setting the long axis of the bone and using the distance from the growth plate.The femoral growth plate is not flat, and we were concerned about errors in distance measurement, so we employed CT data from the tibia. Due to methodological limitations, CT data from the femur and vertebrae were not employed, but the possibility that we could only assess the phenotype of KO mice locally is noted as a limitation in the manuscript.
